# The effect of erythropoiesis‑stimulating agents on lung cancer patients: a meta‑analysis

**DOI:** 10.1007/s10238-024-01391-3

**Published:** 2024-07-05

**Authors:** Zhenhua Tong, Zhumeng Xu, Yaqi Duan, Xue Sun, Bin Qi

**Affiliations:** 1 Department of Research and Training, General Hospital of Northern Theater Command, No. 83, Wenhua Road, Shenyang, 110016 China; 2 Department of Pharmacy, The First Hospital of China Medical University, Shenyang, Liaoning China; 3 Shenyang Pharmaceutical University, Shenyang, Liaoning China; 4 Department of Pharmacy, General Hospital of Northern Theater Command, No. 83, Wenhua Road, Shenyang, 110016 China; 5 Department of Cardiology, National Key Laboratory of Frigid Zone Cardiovascular Diseases (NKLFZCD), General Hospital of Northern Theater Command, No. 83, Wenhua Road, Shenyang, 110016 China

**Keywords:** Lung cancer, Anemia, Erythropoiesis-stimulating agents, Meta-analysis

## Abstract

Previous studies have demonstrated that erythropoiesis-stimulating agents (ESAs) can reduce anemia and improve quality of life in cancer patients, but ESAs may increase mortality. Therefore, we conducted a meta-analysis of randomized controlled trials (RCT) comparing the effect and risk of ESAs about the prevention or treatment of anemia in cancer patients. Four databases including PubMed, Embase, Web of science and Cochrane Library were searched for published RCTS on ESAs in the treatment of anemia in lung cancer patients from 2000 to 2023. Endpoints including mortality, incidence of thrombotic vascular events, blood transfusion requirement, and incidence of adverse events. Our meta-analysis included 8 studies, with a sample size of 4240 patients, including 2548 patients in the ESAs group and 1692 patients in the control group. The risk of mortality was lower in patients using ESAs than control group (RR 0.96, 95% CI 0.92–0.99, *P* = 0.02). But there was no significant difference in the risk of mortality between the patients using ESAs and controls (RR 0.99, 95% CI 0.92–1.06, *P* = 0.69) after removing Pere 2020. Subgroup analysis found that patients diagnosed with small cell lung cancer (SCLC) (RR 1.00, 95% CI 0.92–1.08, *P* = 0.16) or non-small cell lung cancer (NSCLC) (RR 1.01, 95% CI 0.87–1.17, *P* = 0.13) were no significant difference in mortality rate. The thrombotic vascular events increase in patients using ESAs than control group (RR 1.40, 95% CI 1.13–1.72, *P* = 0.002). The blood transfusion requirement of ESAs group was lower than control group (RR 0.56, 95% CI 0.44–0.72, *P* < 0.00001). And the subgroups of Darbepoetin alfa (RR 0.57, 95% CI 0.41–0.79, *P* = 0.003) and Epoetin alfa (RR 0.68, 95% CI 0.47–0.99, *P* = 0.01) had lower transfusion requirements than the control group. In the SCLC subgroup (RR 0.51, 95% CI 0.40–0.65, *P* = 0.34), blood transfusion requirements were lower in the ESAs group, but there was no significant difference between the subgroup of patients with NSCLC (RR 0.61, 95% CI 0.36–1.04, *P* = 0.009). There was no statistically significant difference between the two groups in the incidence of adverse reactions (RR 0.98, 95% CI 0.95–1.00, *P* = 0.10). In conclusion, ESAs does not increase the mortality of lung cancer patients or may reduce the risk of death, and can reduce the need for blood transfusion, although ESA can increase the incidence of thrombotic vascular adverse events.

**Registration** PROSPERO CRD42023463582.

## Introduction

Bone marrow suppression due to chemotherapy and radiation therapy can cause or exacerbate pre-existing anemia, so anemia is a particularly common complication of cancer, which leads to hypoxia of tumor cells, reduces sensitivity to radiation and chemotherapy, and leads to rapid disease progression and reduced quality of life [1]. In the European Cancer Anemia Survey (ECAS), a 39-month follow-up of 15,367 patients received chemotherapy found that the incidence of anemia was 53.7% in cancer patients, 29.3% with mild anemia, and 1.3% with severe anemia, and only 39% of these patients were treated [2]. Therefore, improving anemia will improve the quality of life of cancer patients. Current treatments include blood transfusions, erythropoietin, and iron [1]. For cancer patients, dietary therapies and drugs work more slowly [3], and there is a risk of infection transmission and alloimmunization when they receive red blood cell transfusion [1]. It has been shown that ESAs not only increase hemoglobin concentrations in cancer anemia and reduce the need for red blood cell transfusions, but also improve the quality of life of cancer patients [4]. However, erythropoiesis-stimulating analogs have also been shown to increase mortality by 10% in chemotherapy patients [5], and they have been reported to increase the risk of thromboembolism and may stimulate tumor growth [4].

Erythropoietin (EPO) is a glycoprotein hormone naturally produced by peritubular cells in the kidneys that stimulate the production of red blood cells in the bone marrow [6, 7], and ESAs are recombinant versions of pharmacologically produced EPO. The ESAs currently on the market are epoetin, dabepoetin, and methoxypolyethylene glycol-epoetin β [7]. A study of patients with lung cancer treated with platinum-based chemotherapy showed that 59% of patients in the darbepoetin alfa group died compared with 69% in the placebo group, and the median overall survival was 46 weeks in the darbepoetin alfa group and 34 weeks in the placebo group [8], suggesting a potential survival benefit of ESA treatment in patients with cancer anemia. There are also many meta-analyses that have reported the effect of ESA on the quality of life of patients in the treatment of cancer anemia. Bohlius et al. [9] performed a meta-analysis of survival rates in 42 ESA tumor trials involving 8167 patients that showed no statistically significant increase in the risk of death associated with ESA use. Similar findings were reported in the study by Ross et al. [10] and an updated meta-analysis by Bohlius et al. [11]. However, a large meta-analysis by Bennett et al. (2008) showed that mortality was significantly higher in the ESA group than in the control group. Therefore, there is still much controversy about whether ESAs reduce mortality in cancer patients.

In fact, among all solid tumor types, lung cancer patients have the highest incidence of anemia and transfusion use, with 50–60% of patients developing anemia and 30 to 40% requiring transfusion after four to six cycles of chemotherapy [12]. Therefore, we selected lung cancer patients of anemia for meta-analysis to investigate whether ESAs had an effect on mortality, blood transfusion rates, incidence of thrombovascular adverse events, and adverse events.

## Materials and methods

### Literature search

The English search terms were mainly “lung neoplasms, lung tumor, NSCLC, SCLC, erythropoietin, cancer, and anemia.” The literature on clinical RCTs of ESA for the treatment of anemia in patients with lung cancer published in the PubMed, Embase, Web of science, and Cochrane Library electronic databases from 2000 to 2023 was searched, and a total of 3977 articles were retrieved. We searched for studies that included (1) age > 18 years with a diagnosis of lung cancer, including SCLC or NSCLC; (2) the included studies were RCTs, with or without blinding or allocation concealment, and the included studies must contain valid data and evaluation measures; (3) the experimental group was treated with ESAs (Darbepoetin alfa, Epoetin alfa, Erythropoietin), and the control group was treated with placebo or no placebo, and other anemia-related treatment measures and drug use (blood transfusion, iron therapy, radiotherapy and chemotherapy, etc.) were consistent between the two groups. The main exclusion criterion were (1) primary hematologic disorders known to cause anemia; (2) unstable or uncontrollable disease or heart condition related to or affecting cardiac function; (3) other known primary malignancies; (4) Unstable or uncontrolled comorbidities such as diabetes mellitus or hypertension; (5) have received at most any erythropoietin therapy in the last 8 weeks.

### Data extraction and quality assessment

Two review authors independently searched and screened the literature, resolved by discussion if there were differences. And after excluding trials that clearly did not meet the inclusion criteria, the full text was read for those that may have met the inclusion criteria to determine whether the inclusion criteria were met. The extracted data mainly included (1) basic information of the included studies: study title, first author, nationality, date of publication, duration of follow-up, and source of the literature; (2) characteristics of the studies: general conditions of the study subjects, baseline comparability of patients in each group, sex ratio of the patients, average age of the patients, interventions, and drug dosages; (3) outcome measures: mortality rate, transfusion rate, incidence of adverse events, vascular thrombotic events incidence; (4) key elements of risk of bias evaluation. The methodological quality of the included studies was assessed independently by 2 investigators, and the risk of bias of the included studies was assessed using the risk of bias assessment tool for randomized controlled trials recommended by the Cochrane Handbook, including generation of randomized sequences, concealment of allocations, double-blinding of implementers and participants, blinding in outcome assessment, incomplete outcome data, selective publication, and other biases. And we used Review Manager 5.4.1 software to create quality evaluation charts.

### Statistical methods

Meta-analysis of the final included literature was performed using Review Manager 5.4.1 statistical software, and relative risk ratio (RR) and 95% confidence interval (CI) were selected as effect indicators for dichotomous variables. Heterogeneity between included studies was judged in conjunction with *I*^2^: values of *I*^2^ > 50% indicate significant heterogeneity, in which case a random-effects model was used and further sensitivity analyses were performed and sources of heterogeneity were explored using Stata 14.0 or subgroup analyses. Otherwise a fixed effects model was used. If there was statistical heterogeneity between studies (*P* < 0.1, *I*^2^ > 50%), the source of heterogeneity was analyzed, and subgroup analyses were performed for factors that might have contributed to the heterogeneity, and a random-effects model was used if there was statistical heterogeneity without clinical heterogeneity between the two study groups or if the difference was not statistically significant. If the heterogeneity originated from low-quality studies, sensitivity analysis was performed. Descriptive analysis was used if the heterogeneity between the two groups was too large or if the data source could not be found.

## Results

### Literatures searching results

**Fig. 1:**
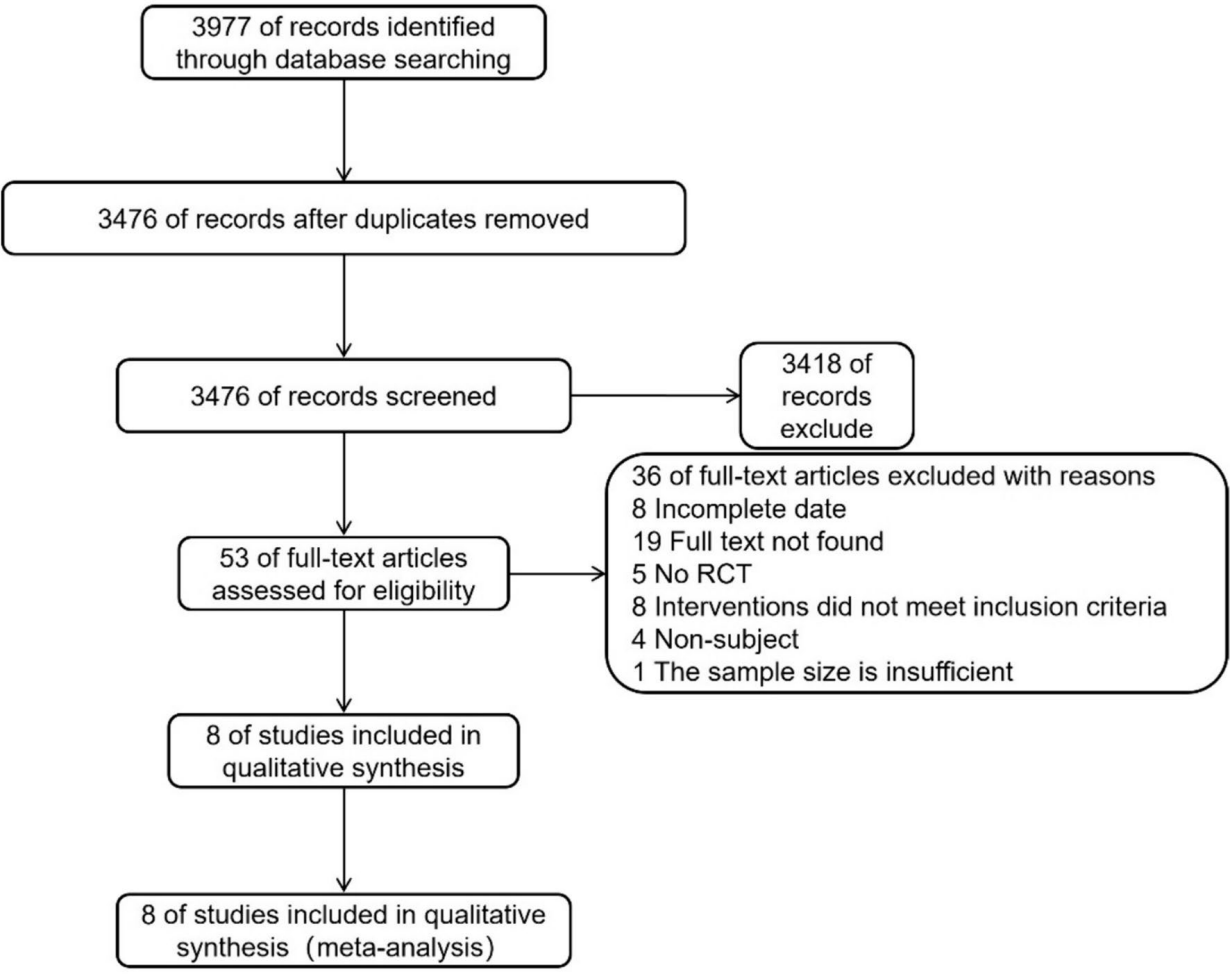
Flowchart for inclusion of literature

PubMed, Embase, Web of science, and Cochrane Library were used to search for publicly available RCTs, and a total of 3977 relevant literature were retrieved; excluding duplicates, 3476 articles remained. By reading the titles and abstracts, 53 articles were left. By reading the full text, a total of 8 papers were finally included for meta-analysis (Fig. 1).

### Basic information about the included literature

The 8 included studies [8, 13–19] were published in English between 2000 and 2020, with a total sample size of 4240 cases, including 2548 cases in the experimental group and 1692 cases in the control group. The main study population of 3 studies published by Pere, Jürgen and James R, were NSCLC patients, and 4 studies published by Sylke, Robert, Hye-Suk and Thomas were SCLC patients; Johan’s main study population included NSCLC patients and SCLC patients. Four studies used darbepoetin alfa, 3 studies used epoetin alfa, and 1 study used RCHT+EPO. By organizing and summarizing the baseline data of the 8 included studies, the baseline characteristics were basically balanced between the experimental and control groups. Following is the basic information and basic characteristics of the included studies (Tables 1 and 2).

**Table 1:** Basic information on included studies

Study	Country	Gender		Disease type		ECOG score			Research type
		Experimental group(male/female)	Control group (male/female)	NSCLC (experimental group/control group)	SCLC (experimental group/control group)	0–1 (experimental group/control group)	2 (experimental group/control group)	3 (experimental group/control group)	
Pere 2020	Spain	1103/577	557/279	1680/836	0/0	2069/831	7/5	0/0	RCT
Jürgen 2014	Germany	153/42	145/45	195/190	0/0	0/0	0/0	0/0	RCT
Sylke 2011	Germany	25/12	24/13	0/0	37/37	35/34	2/3	0/0	RCT
Robert 2008	Austria	187/111	198/100	0/0	298/298	233/235	65/63	0/0	RCT
Hye-Suk 2008	Korea	33/19	7/2	0/0	40/21	36/21	4/0	0/0	RCT
James R.2007	Canada	17/16	20/17	33/37	0/0	14/20	0/0	0/0	RCT
Thomas 2005	America	59/50	64/51	0/0	109/116	73/83	34/32	1/0	RCT
Johan 2002	Belgium	111/45	116/42	108/114	48/44	131/121	24/37	1/0	RCT

**Table 2:** Basic characteristics of included studies

Author	Year	Country	Follow-up time	Disease	Age	Intervening measure	Control group	Sample capacity	Outcome indicator
Pere	2020	Spain	36 month	NSCLC	I: 62.0 C: 63.0	Darbepoetin alfa	Placebo	2516	OS,PFS,Mortality, Adverse event
Jürgen	2014	Germany	72.6 month	NSCLC	I: 61.8 C: 63.5	RCHT + EPO	RCHT	385	OS,PFS,Mortality,Adverse event,Blood Transfusion requirement
Sylke	2011	Germany	24 month	SCLC	I: 61 C: 59	Carboplatin/Etoposide + Darbepoetin alfa	Carboplatin/Etoposide	74	OS,PFS,Adverse event,Blood transfusion requirement,QOL
Robert	2008	Austria	12 month	SCLC	I: 60.6 C: 61.3	Darbepoetin alfa	Placebo	596	Adverse event,Blood transfusion requirement,OS,Hb
Hye-Suk	2008	Korea	25.3 month	SCLC	I: 63 C: 63	Epoetin alfa	Untreated	61	Hb,Adverse event
James R	2007	Canada	16 month	NSCLC	I: 68 C: 70	Epoetin alfa	Placebo	70	OS,Hb,QOL
Thomas	2005	America	36 month	SCLC	I: 64.4 C: 63.2	Epoetin alfa	Placebo	224	Hb,Blood transfusion requirement,Adverse event
Johan	2002	Belgium	12 month	SCLC and NSCLC	I: 61.6 C: 61.3	Darbepoetin alfa	placebo	314	Blood transfusion requirement,Adverse event,OS,Mortality

### Results of risk of *bias* assessment

**Fig. 2:**
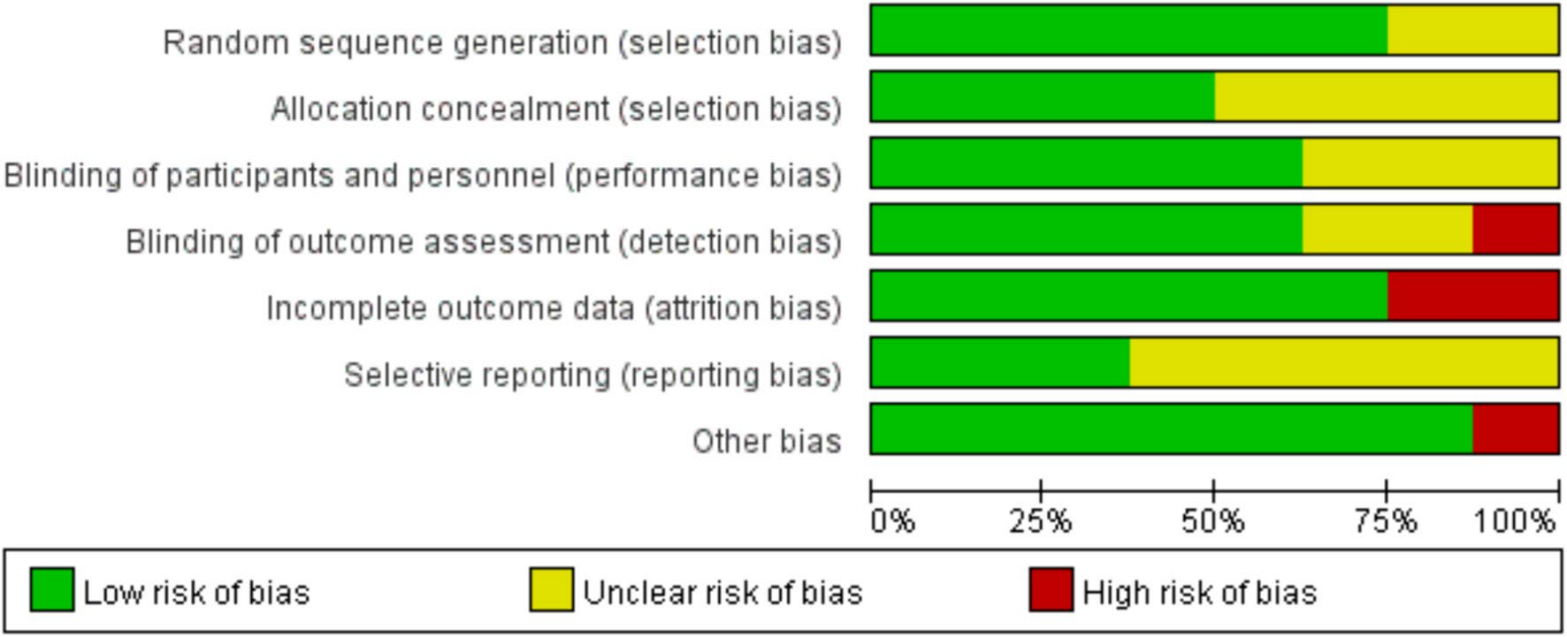
Risk of bias evaluation of the included literature

An assessment of the methodological quality of the included studies is shown in Table 3. For the quality assessment of the literatures, most of the assessed entries were identified as low risk, which suggested no significant risk of bias (Fig. 2).

**Table 3:** Methodological quality assessment of included studies

Author (year)	Random sequence generation (selection bias)	Allocation concealment (selection bias)	Blinding of participants and personnel (performance bias)	Blinding of outcome assessment (detection bias)	Incomplete outcome data (attrition bias)	Selective reporting (reporting bias)	Other bias
Pere 2020	+	+	+	+	+	+	+
Jürgen 2014	?	?	?	−	+	?	+
Sylke 2011	+	?	?	+	−	+	+
Robert 2008	+	+	+	?	+	+	+
Hye-Suk 2008	?	?	?	?	+	?	−
James R. 2007	+	+	+	+	−	?	+
Thomas 2005	+	?	+	+	+	?	+
Johan 2002	+	+	+	+	+	?	+

### Data analyses

#### Mortality

**Fig. 3:**
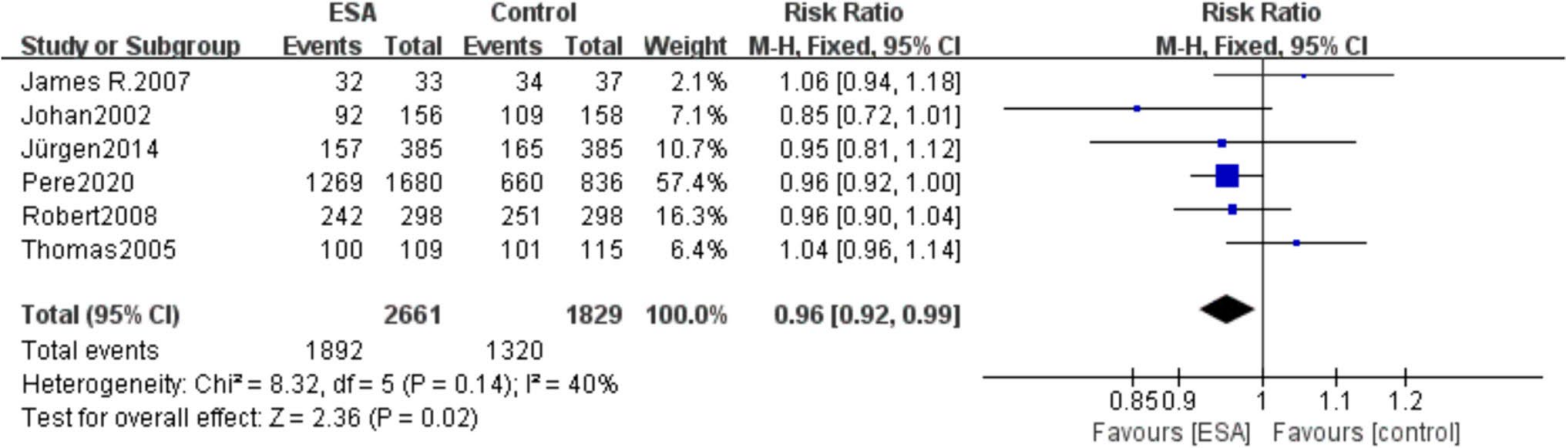
Forest plot of total mortality between erythropoietin and control groups

A total of 6 included studies (*I*^2^ = 40%, *P* = 0.14) reported mortality of patients from the start of treatment to the end of study follow-up, with 1892 (71.10%) of 2661 patients in the erythropoietin group and 1320 (72.17%) of 1829 patients in the control group. The result showed that the risk of mortality was lower in patients using ESAs than the control group (RR 0.96, 95% CI 0.92–0.99, *P* = 0.02) (Fig. 3).

**Fig. 4:**
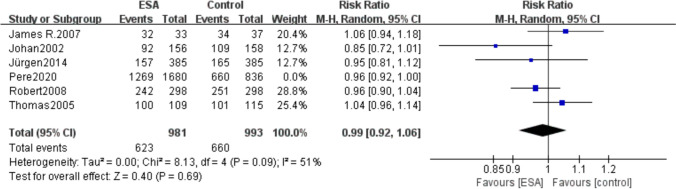
Forest plot of total mortality between the erythropoietin group and the control group after exclusion of the literature with a larger proportion of the sample size

We removed data of the Pere 2020 which had a larger sample size, and there was no significant difference in mortality risk between patients treated with ESAs and control groups (RR 0.99, 95% CI 0.92–1.06, *P* = 0.69) (Fig. 4).

**Fig. 5:**
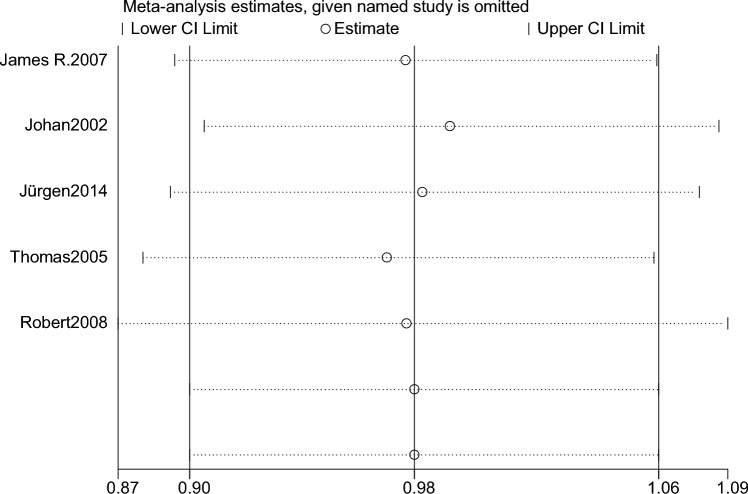
Mortality sensitivity analysis

By using Stata 14.0 statistical software for sensitivity analysis of the 5 included studies, the main indicators of each group were excluded one by one analysis, and the final results did not change much, indicating that the present results are reliable (Fig. 5).

**Fig. 6:**
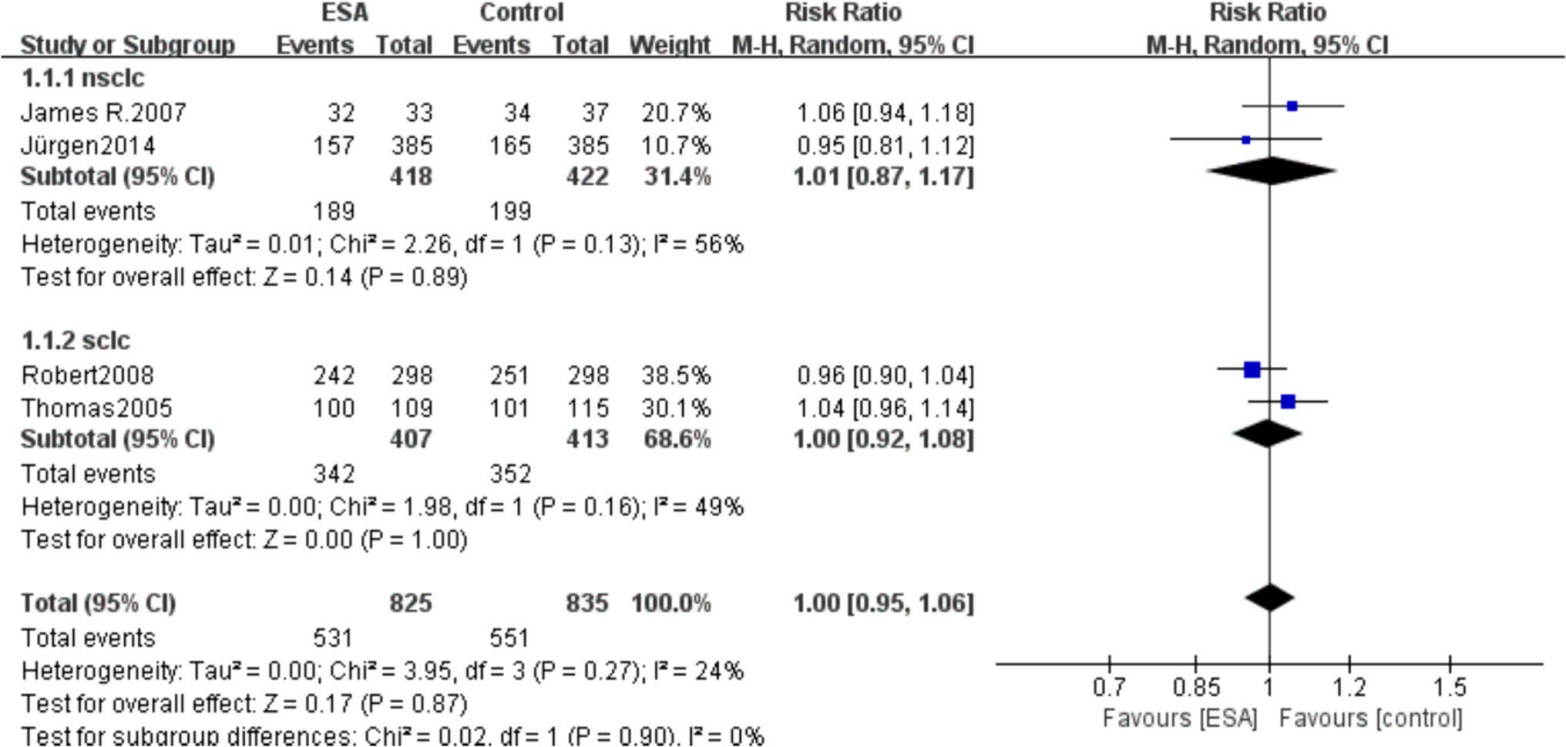
Mortality subgroup analysis—NSCLC and SCLC forest plots

Among the included studies, 2 studies included patients with NSCLC and 2 included SCLC, so we did subgroup analyses based on lung cancer type. And the results showed no significant difference in the risk of mortality between the NSCLC subgroup (RR 1.01, 95% CI 0.87–1.17, *P* = 0.13) and SCLC subgroup (RR 1.00, 95% CI 0.92–1.08, *P* = 0.16) (Fig. 6).

### Incidence of thrombotic vascular events

**Fig. 7:**
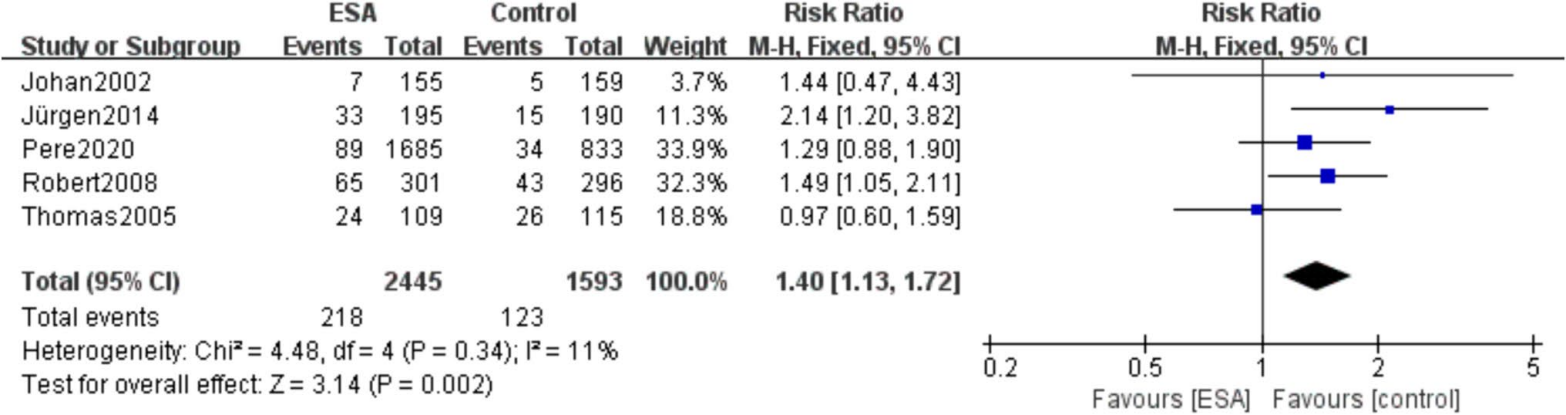
Forest plot of the incidence of thrombovascular adverse events between the erythropoietin and control groups

A total of 5 included studies (*I*^2^ = 11%, *P* = 0.002) reported the occurrence of thrombotic vascular adverse events, including 218 (8.9%) of 2445 patients in the ESAs group and 123 (7.7%) of 1593 patients in the control group. The result showed that the incidence of adverse thrombovascular events was higher in patients using ESAs than in the control group (RR 1.40, 95% CI 1.13–1.72, *P* = 0.002) (Fig. 7).

### Blood transfusion requirements

**Fig. 8:**
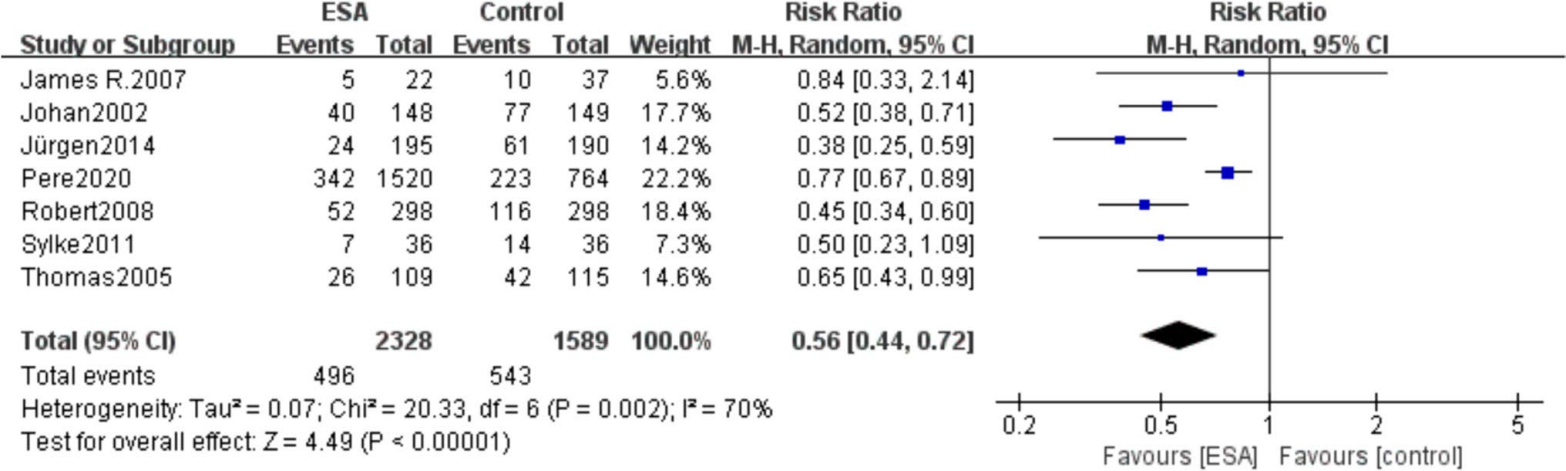
Forest plot of blood transfusion requirements between the erythropoietin group and the control group

A total of 7 included studies (*I*^2^ = 70%, *P* = 0.002) reported patients with transfusion requirements during treatment, including 496 (21.3%) of 2328 patients in the ESAs group and 543 (34.17%) of 1589 patients in the control group. Our studies showed that patients in the ESAs group had lower transfusion requirements than those in the control group (RR 0.56, 95% CI 0.44–0.72, *P* < 0.00001) (Fig. 8).

**Fig. 9:**
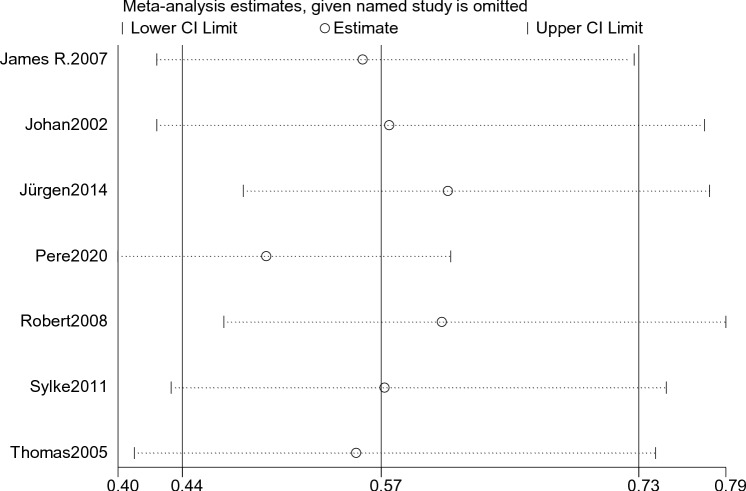
Sensitivity analysis of blood transfusion requirements

After sensitivity analysis of the 7 included studies by using Stata 14.0 statistical software, the results of the sensitivity analysis showed that the 2 included studies that published by Jürgen 2014 and Robert 2008 had a large impact on the results, and the study of Pere 2020 had the largest impact on the results (Fig. 9).

**Fig. 10:**
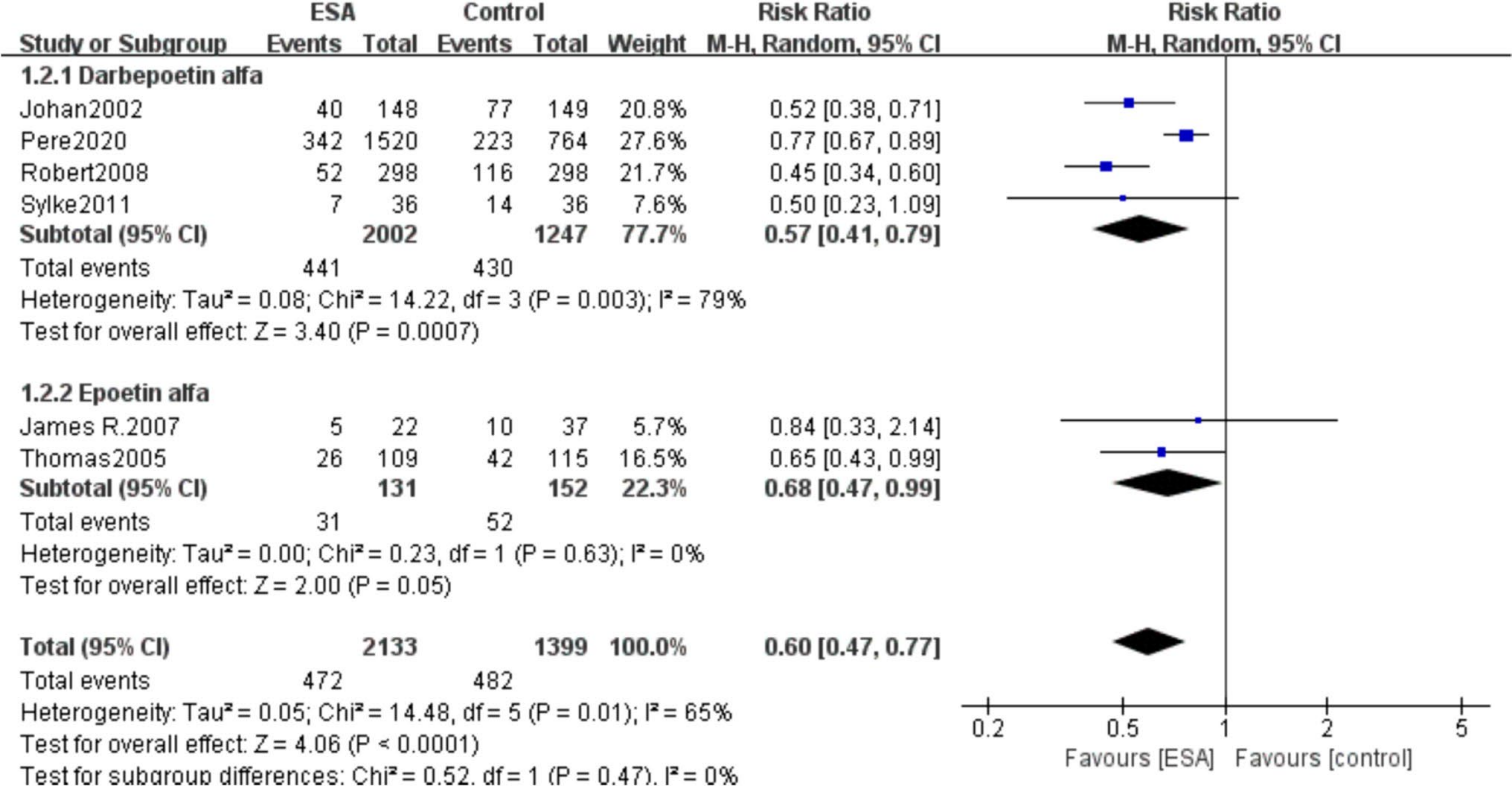
Subgroup analysis of transfusion requirements—forest plot using different types of erythropoietin

A total of 6 studies were included, with 4 studies used Darbepoetin alfa and 2 studies used Epoetin alfa. And the results showed that patients treated with ESAs had lower blood transfusion requirements than control group in both the Darbepoetin alfa group (RR 0.57, 95% CI 0.41–0.79, *P* = 0.003) and the Epoetin alfa group (RR 0.68, 95% CI 0.47–0.99, *P* = 0.01) (Fig. 10).

**Fig. 11:**
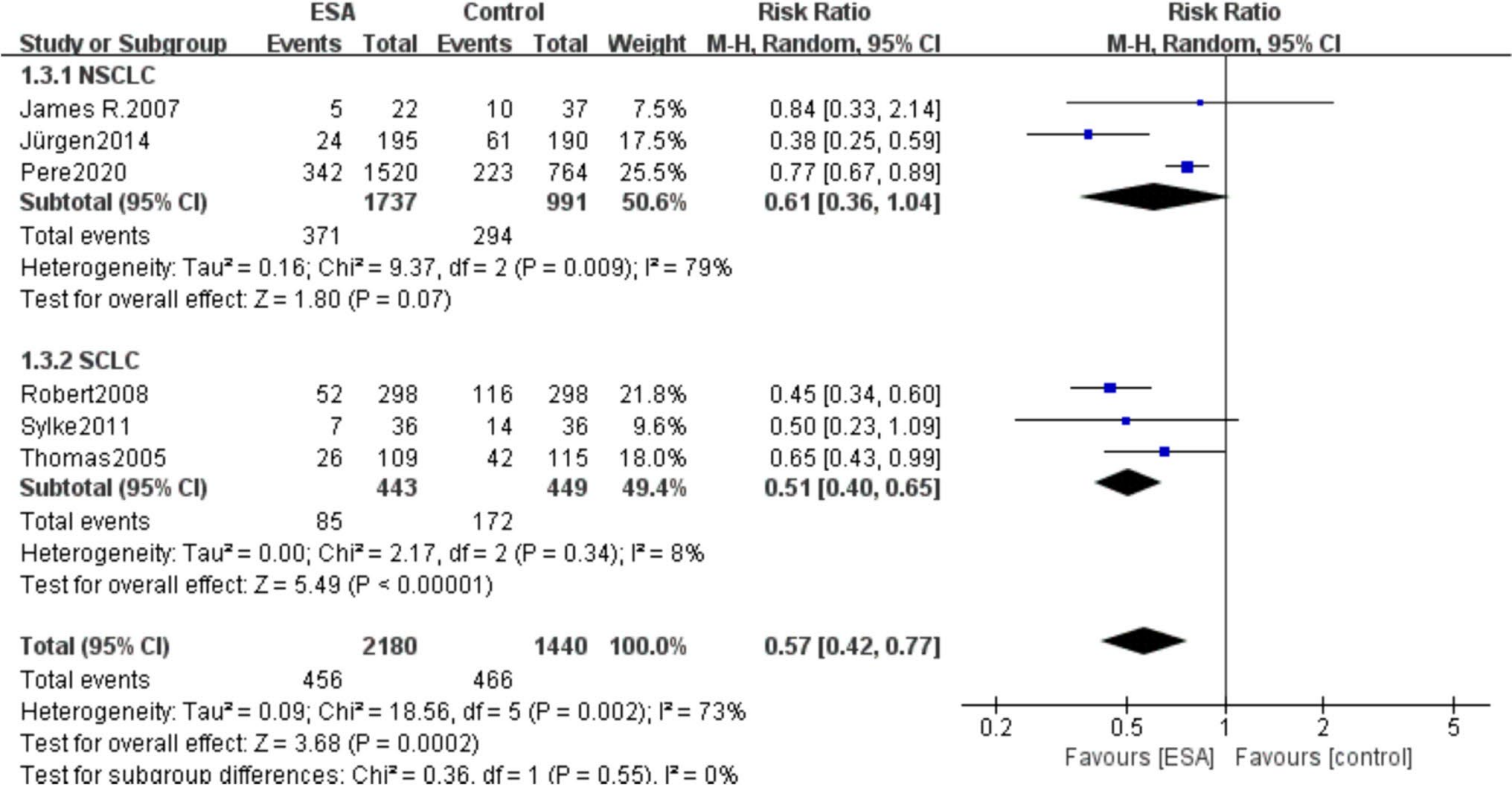
Subgroup analysis of blood transfusion requirements—NSCLC and SCLC forest plots

A total of 6 studies we included, of which 3 included NSCLC and 3 included SCLC, so we did subgroup analyses based on lung cancer type. And the results showed that in the NSCLC subgroup (RR 0.61, 95% CI 0.36–1.04, *P* = 0.009), there was no significant difference in the transfusion requirements between the ESAs group and the control group; whereas, the transfusion requirements in the ESAs group were lower (RR 0.51, 95% CI 0.40–0.65, *P* = 0.34) than those in the control group about the SCLC subgroup (Fig. 11).

### Incidence of adverse events

**Fig. 12:**
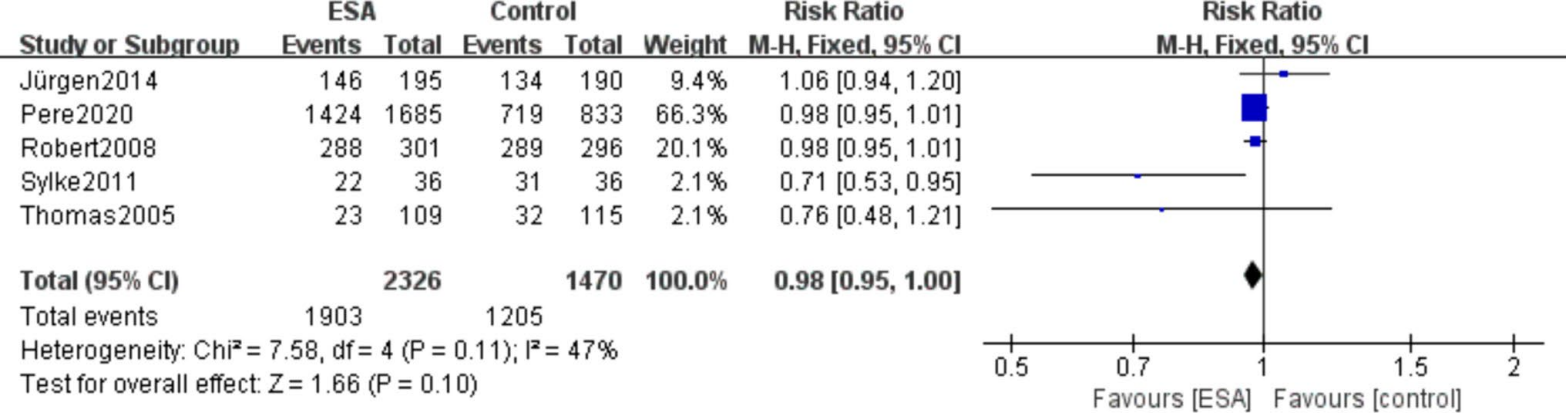
Forest plot of the incidence of adverse events between the erythropoietin group and the control group

A total of 5 included studies (I^2^ = 47%, *P* = 0.11) reported that patients had experienced adverse events during treatment, including 1903 (81.8%) of 2326 patients in the ESAs group and 1205 (81.9%) of 1470 patients in the control group. The results showed that the difference of the two measures between the erythropoietin and control groups was not statistically significant (RR 0.98, 95% CI 0.95–1.00, *P* = 0.10) ( Fig. 12).

## Discussions

Anemia is a common complication in patients with solid tumors, and the etiology of cancer-related anemia is multifactorial, ranging from direct effects of cancer such as tumor hemorrhage and direct effects of bone marrow invasion, results of cancer treatment itself such as chemotherapy, radiotherapy, tyrosine kinase inhibitors (TKIs), and monoclonal antibodies-induced cell death, or chemical factors produced by cancer such as autoantibodies, inflammatory cytokines that affect erythropoietin production and block iron metabolism [20, 21]. Following the cloning of the EPO gene in 1984, EPO was approved for clinical use by the FDA in 1989 [22]. Currently, EPO and its analogs ESAs are mainly used for the treatment of anemia in chronic renal failure and malignancy. Numerous clinical trials have shown [20] that the efficacy and safety equivalence of different ESAs can reduce the need for blood transfusions in patients with chemotherapy-induced anemia, and improve blood flow and quality of life. However, since 2005, the use of these drugs has decreased substantially as data related to declining survival rates have been published [20].

The study by Jürgen et al. [18] showed that no significant deleterious effect of ESAs on short or long-term survival in patients with NSCLC was found at a median follow-up of nearly one year. The results of the Sylke et al. [17] study suggested that the use of Darbepoetin alfa in chemotherapy is still useful in some cases, so the efficacy in avoiding blood transfusions and improving quality of life does not appear to be overshadowed by shortcomings in terms of thromboembolic safety. Previous studies in lung cancer patients receiving platinum-based chemotherapy [8] showed a potential survival benefit in SCLC patients receiving Darbepoetin alfa compared with placebo, with 59% of patients in the experimental group dying compared with 69% in the control group, consistent with our findings on mortality. However, studies by James et al. [15] showed a reduced overall survival rate in patients treated with EPO in patients with advanced non-small cell lung cancer.

In addition, a meta-analysis of survival and other safety outcomes studies on the use of ESAs published in 2010 [23] showed that ESAs did not lead to an increase in mortality in cancer patients. In a pooled analysis of individual-level data from a randomized, double-blind, placebo-controlled trial of Darbepoetin alfa in patients with chemoanemia [24], ESAs had no effect on mortality risk or disease progression. A meta-analysis of the effects of ESAs on lung cancer patients published in 2012 [25] showed that treatment with ESAs reduced transfusion rates and had no effect on overall survival. In a meta-analysis of randomized trials of recombinant human erythropoietin and mortality in cancer patients [26], ESAs increased mortality in all cancer patients, and a similar increase in mortality may occur in chemotherapy patients.

In terms of the efficacy of anemia, the results of most studies appear to be similar. Pere et al. [13] demonstrated in experiments with patients with NSCLC that ESAs increased hemoglobin and reduced the need for blood transfusions in patients with lung cancer or other cancers undergoing chemotherapy without increasing mortality or disease progression. Therefore, for supportive care of patients with lung cancer anemia, there is good evidence that the benefits of using ESAs actually outweigh the possible risks. The results of Robert et al. [8] did not show an increase in survival after treatment with ESAs; however, they reinforced the benefits of ESAs in reducing blood transfusions. The study by Hye-Suk et al. [16] showed that Epoetin alfa was effective in preventing severe anemia during CCRT in patients with limited disease small cell lung cancer (LD-SCLC). The results of Thomas et al. [14] showed that ESAs did not affect tumor response to chemotherapy or survival in patients with newly diagnosed small cell lung cancer.

## Limitations

Our study has some limitations. Firstly, the studies we included may have selection bias, performance bias and measurement bias, etc., which affected the quality of the trials. In addition, different studies did not agree on the measurement time of the same indicator, which led to some measurement bias. Second, patients who used ESAs had a lower risk of in mortality outcomes compared to than controls, which contradicted the findings of previously published studies. We believe that this may be due to differences in the design and reported endpoints of the studies, mortality and disease progression are often only safety endpoints, inconsistent criteria for disease progression, different patient conditions, and different minimum survival times. While different hemoglobin thresholds have been used as the basis for initiation of treatment for ESAs, studies have varied in the management of iron deficiency, which may affect mortality and disease progression, but have not been systematically analyzed due to limitations inreporting. Therefore, after the exclusion of Pere2020, the largest sample size study, the results of five studies showed no significant difference in mortality risk between patients treated with erythropoietin and control groups. Thirdly, in the meta-analysis, we considered the potential for cross-over in the included studies. This phenomenon could introduce bias, affecting the accurate assessment of treatment effects and consequently influencing the accuracy and reliability of the study results. Despite our efforts to control for this situation in the analysis, its impact cannot be completely eliminated. Fourthly, lung cancer exhibits various subtypes and molecular characteristics, leading to different tumor responses to treatment, thereby affecting the consistency and generalizability of the study results. Non-small cell lung cancer includes several subtypes such as adenocarcinoma, squamous cell carcinoma, and large cell carcinoma, each with distinct pathological and molecular features. However, due to limitations in the included study data and variations in study designs, the differential effects of erythropoietin treatment among different subtypes were not comprehensively assessed. In the meta-analysis, we only conducted the subgroup analyses for non-small cell lung cancer and small cell lung cancer, without fully considering the distribution of tumor subtypes in different studies to evaluate differences in the effects of erythropoietin treatment among different subtypes. Meanwhile, the treatment regimen for lung cancer depends on factors such as tumor type, stage, overall health status of the patient, and clinical judgment of the physician, leading to significant differences in treatments received by patients in different studies. Although we attempted to control for this variability in the analysis, for example, by conducting subgroup analyses or sensitivity analyses, there still exist potential confounding factors. Therefore, when interpreting and applying the study results, we must carefully consider the impact of treatment allocation and integrate other evidence from clinical practice to comprehensively assess the reliability of the results. In conclusion, we believe that more high-quality, large sample, multicenter, fully randomized, double-blind controlled clinical trials are needed to demonstrate whether erythropoietin has an effect on the survival of lung cancer patients, so as to obtain more valuable meta-analysis results.

## Conclusions

Our meta-analysis suggests that patients treated with ESAs have a lower risk of death than controls, and the exclusion of one large RCT study and sensitivity analysis and subgroup analysis further demonstrated that ESAs does not increase mortality in lung cancer patients. For patients with lung cancer, especially small cell lung cancer, ESAs may reduce the need for blood transfusion. These benefits are not accompanied by a significant increase in adverse drug events. However, our results also confirm that ESAs increases the incidence of thrombotic vascular events in lung cancer patients. Therefore, it is recommended that lung cancer patients with low risk of thromboembolism should be treated with ESAs to improve the quality of life of patients when anemia occurs.

## Data Availability

No datasets were generated or analysed during the current study.

## Abbreviations

ESAs: Erythropoiesis-stimulating agents
SCLC: Small cell lung cancer
NSCLC: Non-small cell lung cancer
RCT: Randomized controlled trials
EPO: Erythropoietin
RR: Risk ratio
CI: Confidence interval
TKIs: Tyrosine kinase inhibitors
